# A common periodic representation of interaural time differences in mammalian cortex

**DOI:** 10.1016/j.neuroimage.2017.11.012

**Published:** 2018-02-15

**Authors:** Nelli H. Salminen, Simon J. Jones, Gestur B. Christianson, Torsten Marquardt, David McAlpine

**Affiliations:** a UCL Ear Institute, 332 Gray's Inn Road, London, WC1X 8EE, UK; b Brain and Mind Laboratory, Dept. of Neuroscience and Biomedical Engineering, MEG Core, Aalto NeuroImaging, Aalto University School of Science, Espoo, Finland; c Dept of Linguistics, Australian Hearing Hub, Macquarie University, Sydney, NSW 2109, Australia

**Keywords:** Sound source localization, Interaural time difference, Magnetoencephalography, Human, Guinea pig, Auditory cortex

## Abstract

Binaural hearing, the ability to detect small differences in the timing and level of sounds at the two ears, underpins the ability to localize sound sources along the horizontal plane, and is important for decoding complex spatial listening environments into separate objects – a critical factor in ‘cocktail-party listening’. For human listeners, the most important spatial cue is the interaural time difference (ITD). Despite many decades of neurophysiological investigations of ITD sensitivity in small mammals, and computational models aimed at accounting for human perception, a lack of concordance between these studies has hampered our understanding of how the human brain represents and processes ITDs. Further, neural coding of spatial cues might depend on factors such as head-size or hearing range, which differ considerably between humans and commonly used experimental animals. Here, using magnetoencephalography (MEG) in human listeners, and electro-corticography (ECoG) recordings in guinea pig—a small mammal representative of a range of animals in which ITD coding has been assessed at the level of single-neuron recordings—we tested whether processing of ITDs in human auditory cortex accords with a frequency-dependent periodic code of ITD reported in small mammals, or whether alternative or additional processing stages implemented in psychoacoustic models of human binaural hearing must be assumed. Our data were well accounted for by a model consisting of periodically tuned ITD-detectors, and were highly consistent across the two species. The results suggest that the representation of ITD in human auditory cortex is similar to that found in other mammalian species, a representation in which neural responses to ITD are determined by phase differences relative to sound frequency rather than, for instance, the range of ITDs permitted by head size or the absolute magnitude or direction of ITD.

## Highlights

• ITD tuning is studied in human MEG and guinea pig ECoG with identical stimuli.
• Auditory cortical tuning to ITD is highly consistent across species.
• Results are consistent with a periodic, frequency-dependent code.

## Introduction

A sense of space, including the location of objects in the environment, is fundamental to perception. In vision and touch, space is represented at the level of the sensory epithelium—the retina at the back of the eye, and the surface of the skin—and constitutes the major organizational principle of brain centers dedicated to these senses. In contrast, the primary feature represented in hearing—from the cochlea of the inner ear to at least the level of primary cortex—is frequency. To this end, the location of a sound source is computed from information converging from each ear onto neurons in the central nervous system, a process known as binaural (two-eared) hearing. Many species, including humans, make use of two binaural cues, interaural time differences (ITDs) and interaural level differences (ILDs) to perform sound localization with an accuracy of just a few degrees (Mills, 1958). With ITDs of a few 10's of microseconds (millionths of a second) discriminable at the behavioral (Klumpp, 1956, Jeffress and McFadden, 1971, Smoski and Trahiotis, 1986) and neural (Fitzpatrick et al., 1997, Skottun et al., 2001, Tollin and Yin, 2005) levels, brain mechanisms contributing to ITD sensitivity have been of interest since the middle of the 18th century (Thompson, 1877).

**Fig. 1:**
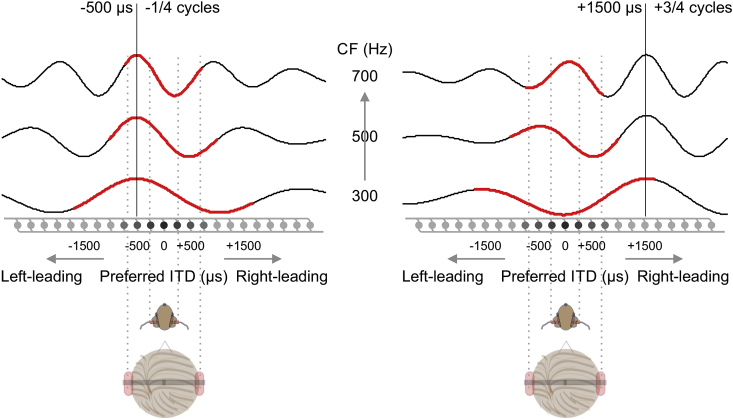
Schematic representation of ITD processing models approximating operations taking place in the auditory brainstem. The neural activation is presented for three frequency channels or center frequencies (CFs) to a noise stimulus with ITDs of −500 μs (left) and +1500 μs (right). The exemplar activation patterns within the three frequency bands are quasi-periodic and scale for sound frequency. According to the classical Jeffress model, this ITD sensitivity is generated by a systematic arrangement of delay lines and coincidence detectors depicted in gray below the activation curves. ITDs beyond the ethological range determined by the size of the head (depicted by gray dashed lines for humans and guinea pigs) are perceived with correct lateralization. Psychoacoustic models account for this by suggesting explicit detectors that encode ITDs over a range considerably greater than the human ethological range, but with a greater density of detectors at smaller ITDs (denoted by change in grey-scale in filled circles). In these models, correct lateralization then comes about from brain mechanisms that favor consistency of activity across frequency channels (straightness, black vertical lines) and that generate a frequency-independent representation of ITD. In contrast, data from small mammals suggest a frequency-dependent representation of ITD in which only ITDs spanning the range ±1/2 cycle of interaural phase (the π-limit - denoted by red part of the curves) are explicitly represented.

For the past 7 decades, research into the neural representation of ITD has been strongly influenced by the classic Jeffress model (Jeffress, 1948), in which ITDs are encoded by an array of coincidence-detector neurons innervated by axons with a systematic arrangement of conduction delays (Fig. 1). This model postulates that inputs arriving at the two ears are converted into a neural map of ITD, with sound frequency represented along the orthogonal axis. The resulting ITD tuning is periodic: in addition to its preferred ITD, coincidence detectors also respond maximally to ITDs at multiple periods of their preferred sound frequency, rendering them sensitive to interaural phase difference (IPD), rather than ITD *per se*. Widely employed instantiations of the Jeffress model (e.g. Stern and Colburn, 1985, Trahiotis and Stern, 1994) posit two additional features in order to account not just for the perceived laterality of sounds—i.e. location along the left-right dimension—in human listeners, but also for the ability to process sounds in the presence of background noise (van der Heijden and Trahiotis, 1999), an important factor in human communication. First, the range of ITD detectors extends well beyond the ethological range of humans (±700 μs, determined by the size of the head; Feddersen et al., 1957, Kuhn, 1977) to account for performance in headphone listening tasks, but the range is weighted for ITDs near zero (referred to as ‘central’ weighting). Second, a computational stage is included to detect the consistency of activity across the orthogonal sound-frequency dimension, in the process converting the periodic, or phasic, representation of primary binaural neurons in the brainstem into an unambiguous representation of ITD that more directly relates to the perceived laterality of the sound.

Nevertheless, despite support from human psychoacoustics and neurophysiological studies in birds, especially in the barn owl—an ITD specialist—(Carr and Boudreau, 1993; Funabiki et al., 2011, Carr et al., 2013), experimental findings in small mammals (McAlpine et al., 2001, McAlpine and Grothe, 2003, McLaughlin et al., 2007) and theoretical considerations (Harper and McAlpine, 2004, Harper et al., 2014) have questioned the form of the neural code for ITD in mammals. In particular, a consistent finding in small mammals is that interaural delays are represented in terms of phase (IPD) rather than time, and supporting the operation of a relative rate code for ITD (e.g. the hemispheric activation ratio), rather than an explicitly coded space map (McAlpine et al., 2001). A key finding from these studies is that the range of internal delays is subject to an upper bound of ½ the period of a neuron's CF—the π-limit (McAlpine et al., 2001, Marquardt and McAlpine, 2007, Franken et al., 2015). Consequently, only ITDs within this range are explicitly represented (red portions of activation curves in Fig. 1), and the range of internal delays is dependent on neural tuning for sound frequency. This questions the currently established models of human ITD processing (e.g. Stern and Colburn, 1985, Trahiotis and Stern, 1994), in which the range of internal delays extends over many cycles of the stimulus period in any one frequency channel (i.e. considerably beyond the π-limit) in order to account for the perception of laterality.

Although *in vivo* physiological studies present a challenge to influential psychoacoustic models of ITD coding, their relevance for understanding human brain function remains unclear, largely due to the difficulties involved in comparing human data to *in vivo* experimental studies. Brain centers responsible for the extraction of binaural cues lie deep in the brainstem, and are difficult to access, even using *in vivo* techniques. Consequently, dictated by the nature of the available recording methods, physiological investigations of spatial hearing in humans are usually made at the level of entire (usually cortical) brain areas, with considerable inference as to the underlying mechanisms that generate ITD sensitivity some three synaptic stages upstream. Further, many experimental paradigms employed in human brain-imaging studies use stimulus parameters that bear little resemblance to those employed in psychoacoustic investigations of the range of internal delays, or are motivated by entirely different questions (for instance, Krumbholz et al., 2005, Johnson and Hautus, 2010, McLaughlin et al., 2015; reviewed in Salminen et al., 2012, Ahveninen et al., 2014). Reconciling the very different data sets across methodologies (psychoacoustics, brain imaging, and electrophysiology) and across species (human and small mammals), therefore, represents a particular challenge. This challenge is further exacerbated by theoretical consideration such as coding efficiency, which suggest at least quantitative differences in the neural representation of ITDs across species based on differences in head size or the sound-frequency range to which an animal is most sensitive (Harper and McAlpine, 2004, Harper et al., 2014, Benichoux et al., 2015). Combined with potential transformations in the neural representation of ITDs along the auditory pathway, these species-specific constraints to spatial listening make it difficult to perform valid comparisons between *in vivo* data from small mammals and brain-imaging or electrophysiological data from humans.

Here, as a first step towards reconciling data from the many different model systems, and towards understanding their implications for ITD processing in the human brain, we examined population-level measures of neural activity—magnetoencephalography (MEG) in humans and electrocorticography (ECoG) in guinea pigs—using stimulus parameters that inform the dominant psychoacoustic models of ITD processing in humans. These parameters elicited a common cortical representation for ITD across the two species, one apparently dominated by the inherent periodicity within different sound-frequency bands. Notably, the substantial difference in head-size between humans and guinea pigs did not appear to be a factor in modulating cortical responses, nor (in humans) did the magnitude of ITDs relative to the ethological range. These data suggest that, to first approximation, periodicity relative to the center frequency of a noise band is an important factor when considering gross cortical activation to sounds containing ITDs. In both species, cortical activity was well explained by a neural model in which the range of internal delays was constrained to the π-limit.

## Materials and methods

### Stimuli

The stimuli were chosen to match previous psychoacoustic investigations that have led to the development of influential models of human ITD perception (Trahiotis and Stern, 1989, Trahiotis et al., 2001). In the first session of MEG recordings and in the guinea pig ECoG, we used a 400-Hz noise band centered at 500 Hz presented with ITDs −1500, −500, +500, and +1500 μs, common parameters in binaural psychoacoustical investigations. Importantly, when imposed on a sound centered at 500 Hz, these ITDs have specific periodic relations: ±500 μs corresponds to ±1/4 periods and ±1500 μs to ±3/4 periods at the stimulus center frequency (see Fig. 3a). The −500 μs and +1500 μs stimuli correspond to the same interaural phase difference (IPD) because −1/4 and + 3/4 are equivalent in phase. ITDs of ±500 μs are within, and ±1500 μs beyond, the human ethological range (±700 μs, Feddersen et al., 1957, Kuhn, 1977). Yet, human listeners perceive all of these to originate from the side of the leading ear. For the guinea pig, all of these ITDs are outside the ethological range (maximally ±330 μs, Sterbing et al., 2003, Greene et al., 2014).

**Fig. 2:**
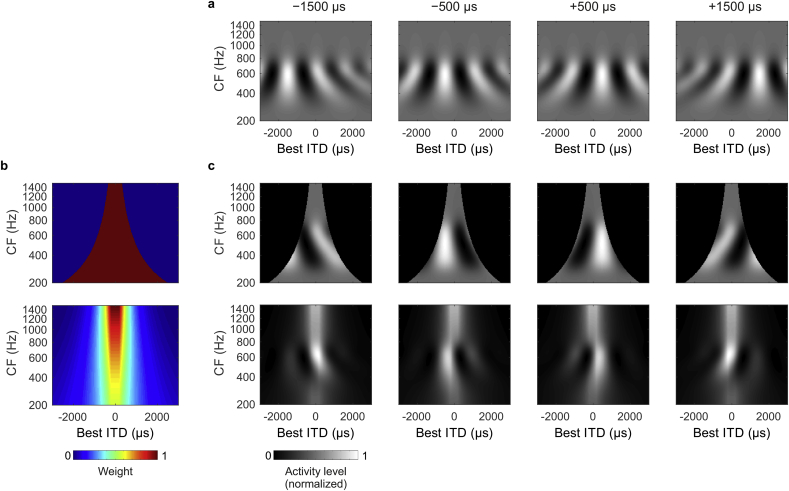
(**a**) Examples of activity patterns in an ITD model. The model activity is depicted over the neurons' center frequencies (CF) and best ITDs to a 400-Hz band of noise centered at 500 Hz and for each of the four stimulus ITDs (−1500, −500, +500, and +1500 μs, from left to right). (**b**) Two distributions of best ITDs were considered: the π-limit (top) and the Stern-Shear distribution, derived by fitting a binaural model to predict a vast amount of psychoacoustic data (bottom). (**c**) Model activity after weighting the four activity patterns in **a** with the two distributions of best ITDs in **b**.

**Fig. 3:**
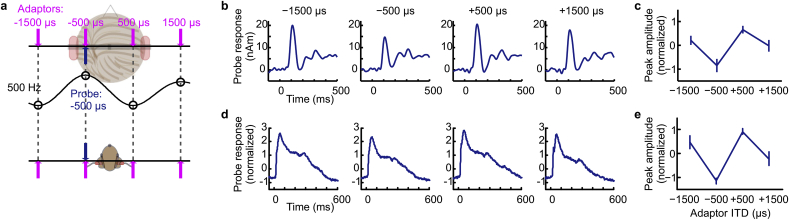
Population-level cortical responses recorded using MEG in humans and ECoG in guinea pigs. (**a**) 400-Hz band-pass noises centered at 500 Hz served as probe and adaptor stimuli. The probe ITD was fixed at −500 μs (blue lines) and the adaptor ITD was either also −500 μs, or separated from the probe ITD in steps of ¼ cycles (purple lines). The black curve illustrates the presumed quasi-periodic ITD tuning curve of the neural population that is most sensitive to the −500-μs probe stimulus. (**b**) Right-hemispheric MEG source waveforms (in nanoampere meter) to the probe sounds in the four adaptor conditions (averaged over 10 subjects). (**c**) Average N1 peak amplitudes ±standard error of the mean (z-scores) of the MEG probe responses. (**d**) Average responses to the probe sound in the four adaptor conditions from ECoG recordings in guinea pigs. (**e**) Average peak amplitudes ± standard error of the mean (z-scores) of the ECoG probe responses.

**Fig. 4:**
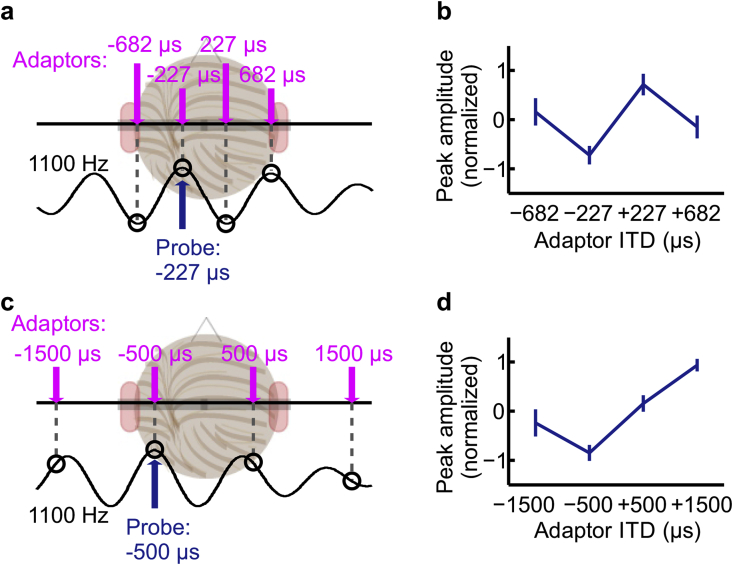
MEG responses to stimuli with 1100-Hz center frequency. (**a**) Periodically related stimulus ITDs corresponding to ±¼ and ±¾ cycle interaural phase (±227 μs and ±682 μs) lie now within the human ethological range. The black line depicts hypothetical activation of the neural population most sensitive to the −227-μs probe sound. (**b**) Average N1 peak amplitudes ± standard error of the mean (z-scores) to the −227-μs probe in the four adaptor conditions in **a**. (**c**) Stimulus ITDs used previously in the 500-Hz centered stimuli. Note that the ITD values (open circles) are unrelated to the quasi-periodicity of the population's ITD tuning curve (black line), which is determined by the 1100-Hz centered stimulus. (**d**) Average N1 peak amplitude ± standard error of the mean (z-scores) to the 1100-Hz −500-μs probe sound in the four adaptor conditions in **c**.

We further wanted to disambiguate the potential effects of periodicity, the operation of a π-limit, and the ethological range on cortical activation to sounds containing ITDs. In the second session of MEG recordings, therefore, we extended the stimulus set to another center frequency, employing a noise band centered at 1100 Hz and increasing the bandwidth to 600 Hz (to ensure correct perception of lateralization to the leading side). Here, two different sets of ITDs were used. First, in order to maintain the same IPD and periodic relations in terms of the stimulus center frequency as for the 500 Hz stimulus, ITDs corresponded to IPDs of ±¼ and ±¾ cycles of the 1100-Hz stimulus period, i.e. ±227 μs and ±682 μs (see Fig. 4a). Importantly, all of these ITDs lie within the human ethological range. The second set consisted of ITDs identical to those used for the 500-Hz stimulus, that is, ±500 μs and ±1500 μs. For the 1100-Hz centered stimulus, these ITDs have no systematic periodic relations, but maintain the same relation to the ethological range as at 500 Hz, the shorter ITDs within, and the longer, beyond the ethological range (see Fig. 4c).

To improve the sensitivity of the population-level recordings to ITD, we used a stimulus-specific adaptation paradigm (Butler, 1972, Salminen et al., 2009, Salminen et al., 2010). The purpose of this paradigm is to ascertain the selective tuning properties of neurons intermingled within the same cortical area. This is particularly important for studying ITD tuning because, in the absence of any topographical representation of space in the mammalian auditory cortex (Werner-Reiss and Groh, 2008), the contributions of differently tuned neurons to the population-level response cannot be segregated in the spatial domain. This paradigm capitalizes on the attenuation of neural responses that an adaptor stimulus induces on the response to a subsequent probe sound (Bartlett and Wang, 2005, Werner-Reiss et al., 2006, Brosch and Scheich, 2008). This attenuation is stimulus-specific, so that an adaptor differing from the probe along a stimulus dimension (here, ITD) generates less attenuation than an adaptor identical to the probe. The strength of the adaptation therefore provides a measure of the overlap between neural populations responsive to the probe and adaptor sounds. Stronger adaptation follows from greater overlap, and weaker adaptation from lesser.

Here, sounds were presented in combinations of probes and adaptors with varying ITDs. The probe ITD was kept constant, whilst that of the adaptor was varied. Neural sensitivity to ITD was then inferred from the extent to which the response amplitude to the probe depended on the ITD of the adaptor. The probe was always the shortest left-leading ITD (−500 or −227 μs), and all four ITDs were employed as adaptors. The magnitude of the ITD-specific adaptation could be expected to reflect the periodic relations between the probe and adaptor ITDs or, alternatively, to depend on their distance in terms of absolute ITD or perceived laterality. For instance, if the periodic relationship between stimulus ITDs is the determining factor, responses to the −500-μs probe at 500 Hz should be least adapted by the +500-μs adaptor because it has the opposite IPD (180°, i.e. of opposing phase). However, if adaptation is determined by absolute ITD or perceived laterality, the weakest adaptation should follow from the +1500 μs adaptor that is the furthest away in terms of ITD, and perceived to originate from the opposite side.

The duration of each noise burst was 200 ms (including cosine-gated onset and offset ramps) for both 500-Hz and 1100-Hz stimuli. The ITD was imposed on the sounds so that the onset and offset were concurrent in the two ears, i.e. only ongoing ITDs were present. The probes and adaptors were presented as alternating pairs with a constant silent inter-stimulus interval (ISI) of 800 ms. The ISI was chosen to fall within a range in which recovery from adaptation is fast, but excessively strong adaptation that might prevent the generation of robust N1 responses is avoided (Sams et al., 1993). Each adaptor-probe combination was presented in a separate stimulation block lasting approximately 5 min. In an additional reference condition, the probes were presented without intervening adaptors. The timescale of the stimulus presentation was similar to that presented in previous studies showing robust location-specific adaptation in cortical responses (Butler, 1972, Salminen et al., 2009, Salminen et al., 2010). Further, the relatively long ISI was such that any adaptation effect is likely to be of cortical origin (Bartlett and Wang, 2005, Werner-Reiss et al., 2006). Therefore, the ITD-specific adaptation here is expected to reflect specifically the properties of cortical neurons.

### MEG recordings in humans

Eighteen volunteers took part in the experiments (age mean 26, standard deviation 5, 4 female), 12 in the first (500-Hz stimulus) and 11 in the second (1100-Hz stimulus) session. Written informed consent was obtained prior to the recordings. The experimental procedures were approved by the Ethical Committee of Aalto University. The data of two subjects in session 1 and one subject in session 2 were discarded due to low signal-to-noise ratio. During the recordings, the subjects were instructed to sit still and to focus on reading a self-selected text.

The recordings were performed with a 306-channel MEG device with 102 pairs of orthogonal planar gradiometers and 102 magnetometers (Vectorview, Elekta-Neuromag, Helsinki, Finland). The data was acquired continuously with sampling rate 1024 Hz and pass-band 0.01–300 Hz. Five head-position indicator coils were attached to the head and their positions were digitized prior to the recordings along with three anatomical landmarks and about 20 additional points along the scalp. Eye-movements and blinks were monitored with horizontal and vertical electro-oculogram. Event-related fields (ERFs) were obtained offline by filtering the data at 1–30 Hz and averaging from 100 ms prior to 500 ms after probe onset. Epochs with deflections larger than 3000 fT/cm in the gradiometers or 150 μV in the electro-oculogram were discarded.

The analysis focused on the amplitude of the N1 response arising from the auditory cortex and peaking at around 100 ms after stimulus onset in the ERFs. The amplitude of the N1 was quantified using equivalent current dipole (ECD) modelling with a spherical head model. The analysis was performed for the two hemispheres separately based on 22 pairs of gradiometers above the temporal lobe. First, an ECD model was obtained using the reference condition in which no adaptors were presented by fitting an ECD at 1-ms intervals to the ERF. A peak in source strength within the 70–150 ms post-stimulus time window with goodness of fit exceeding 80% was identified and the corresponding coordinates were used as the N1 model for the remaining conditions. The location and orientation of the N1 model were kept constant and the source strength was allowed to vary. The N1 amplitude was identified as a peak in the resulting source wave at the 70–150 ms latency. As is typically observed in MEG recordings, responses in the left auditory cortex were considerably weaker than in the right. Here, this tendency was further strengthened by the probe sound being perceived ipsilateral to the left hemisphere. The variation of N1 amplitude in the left hemisphere did not reach significance in any of the stimulus conditions and therefore all results reported here concern the right hemisphere.

### ECoG recordings in guinea pigs

Experiments were carried out in accordance with the Animal (Scientific Procedures) Act of 1986 of Great Britain and Northern Ireland under license number PPL 70/06826. Adult tricolour guinea pigs were anaesthetised with urethane (1.5 g.kg-1 in a 20% solution), buprenorphine (0.075 mg.kg-1), carprofen (4 mg.kg-1) and dexamethasone (20 mg.kg-1), tracheotomised and placed onto a homeothermic heating pad to maintain core body temperature. Bronchial secretions were suppressed with atropine sulphate (0.2 ml of a 0.6 mg.ml-1 solution) and additional analgesia was provided when necessary with fentanyl (0.15–0.315 mg.ml-1). To expose the auditory meatus, the tragus was excised and to maintain pressure equalisation across the tympanum the auditory bullae were vented bilaterally. Stimuli were delivered via hollow ear bars containing ER-4 MicroPro earphones (Etymotics), driven by an RME Fireface UC audio interface, and were identical to those used in human studies.

To expose the cortex, an incision was made along midline and the temporalis muscle removed from the recording site. A craniotomy and durectomy exposing primary auditory cortex (A1) allowed placement of a 16-channel NeuroNexus surface array, permitting recording of the Electro-Corticogram (ECoG). The array was positioned under visual guidance onto the temporal lobe, as near to the edge of the pseudo-sylvian sulcus as possible to maximise coverage of low-frequency A1. Correct placement was confirmed by measuring frequency tuning and the tonotopic gradient across the surface array.

### Computational model

We tested the ability of a simple cross-correlation model to account for the data for both species. This model consists of three stages. In the first stage, noise stimuli are filtered through a pseudo-cochlea [a bank of 500 equivalent-rectangular-bandwidth (ERB) (Moore, 1983) Gammatone filters between 200 Hz and 1.5 kHz] to model processing by the peripheral auditory system. In the second stage, activity of neurons in each frequency channel is modeled in the form of a binaural cross-correlation function. This simulates the activation of neurons with positive and negative best delays within each frequency channel. This is similar to the delay and coincidence detection mechanisms suggested in the classic Jeffress model and generates a neural map of ITD vs. frequency. Examples of these maps are presented in Fig. 2a for the 500-Hz stimulus. Here, only negative delays (i.e. leading at the contralateral, left, ear) were included to model the preference for contralateral ITDs in each brain hemisphere. In the third, adaptation stage of the model, the influence of the adaptors with various ITDs on responses to the probe is modelled by generating an activation pattern (i.e. cross-correlogram) for each adaptor, normalizing its binaural cross-correlation functions to this adaptor between [0,1] and inverting it (1-x). This adaptation function is then multiplied by the activation pattern of the probe, to produce the adapted probe response. Finally, to produce a population-level prediction for the probe response amplitude, the activity is averaged across best ITDs and center frequencies. Each model condition was repeated 10 times with independent samples of the noise stimulus.

In order to explore the potential dependence of the model predictions on the precise distribution of best ITDs, we applied different weights to the cross-correlogram (i.e. the ITD-frequency map) to simulate greater or lesser representation of neurons at different combinations of best frequency and best ITD (Fig. 2b). Two distributions were considered. First, for a model distribution constrained to the π-limit, the distribution was homogenous within these limits. Second, the Stern-Shear distribution (Stern and Shear, 1996) was included as a representative of the class of models generated by psychoacoustic observations in which the weights of best ITDs decline with increasing magnitude of delay, emphasizing the activation of “centrally” located neurons near zero best ITD, but also includes neurons with best ITDs beyond the π-limit. These psychoacoustic models include a stage of second-order coincidence detection (or straightness weighting) that detects the consistency of ITD information across frequencies, but here these further stages were not included because they have not been defined to the level of detail necessary for formulating physiological predictions.

### Statistical analyses

The statistical significance of ITD-specific adaptation in both human MEG and guinea pig ECoG data was tested with repeated-measures ANOVA on the probe response amplitudes with the four adaptor conditions as repeating factor, for each probe condition separately. These were followed by post-hoc planned comparisons testing two alternative hypotheses: periodicity-based code with weights 1, -1, 1, and −1 and laterality-based code with weights −1, −1, 1, and 1 (given to the four adaptors in left-to-right order). The fit of the model predictions to the data was also tested with planned comparisons. The average model output for each adaptor condition was used as weights. To fulfill the requirement of the statistical test for weights that average to zero, the outputs were first scaled by subtracting the mean across the four adaptor conditions. For illustration, the response amplitudes were normalized separately for each subject by subtracting the mean and dividing by the standard deviation across the adaptor conditions (i.e. by converting them into a z-score). These normalized values were used for illustration only—all statistical analyses were performed on the original absolute values.

## Results

### A common periodic representation of ITDs in human and guinea-pig cortex

We first assessed the cortical representation of ITD in human listeners and in guinea pigs for band-pass noise stimuli centered at 500 Hz. We employed an adaptation paradigm in which the ITD of the probe sound was fixed at −500 μs (leading on the left by ¼ cycle of the center frequency), and the ITD of the adaptor sound set to either −1500 μs (leading on the left by ¾ cycle), −500 μs (i.e. identical to the probe), +500 μs (leading on the right by ¼ cycle) or +1500 μs (leading on the right by ¾ cycle, Fig. 3a). All of these stimuli are perceived by human listeners as originating from the side to which the sound is leading in time (Stern et al., 1988; Trahiotis and Stern, 1989, Yost et al., 2007), even though ITDs of ±1500 μs lie far beyond the ethological range of ITDs in humans.

In MEG, the peak amplitude of the N1 response to the −500-μs probe was modulated by the ITD of the adaptor (Fig. 3b–c; repeated-measures ANOVA with adaptor condition as repeating factor, F[3,27] = 3.34 p = 0.034). Responses to the probe were smallest when the ITD of the adaptor and probe were identical, i.e. both were −500 μs, and largest when the adaptor was +500 μs, i.e. having the opposite IPD, and also leading at the opposite ear to the probe. The ±1500-μs adaptors generated probe responses of intermediate magnitude. Thus, despite being least like the −500-μs probe—in terms of its distance from the probe in ITD space and in terms of its lateralization percept—the +1500-μs adaptor did not generate the largest release from adaptation. The pattern of data is consistent, however, with the hypothesized periodic activation pattern in a neural population tuned to the 500-Hz center frequency and ITD of the probe stimulus (illustrated in Fig. 3a): the greater the activation by the adaptor ITD in this curve, the smaller the response amplitude to the probe. Two planned comparisons were performed to account for the variation in N1 amplitude. The first comparison determined how well the data could be explained by the relationship between the adaptor and probe ITD with respect to the stimulus center-frequency, i.e. strong adaptation was predicted for adaptors of −500 μs and +1500 μs (weight of −1 in the comparison) and weaker adaptation for +500 μs and +1500 μs (weight of +1 in the comparison). This contrast was significant (p = 0.012) though note that, numerically at least, the data deviate somewhat from what would be predicted based on responses being fully determined by the periodic relations (i.e. response amplitudes for adaptor conditions −1500 and + 500 and for −500 and + 1500 were not equal). The second comparison tested whether the N1 amplitude could be explained as a function of perceived side of laterality with strong adaptation following from −1500 μs and −500 μs adaptors (weight of −1) and weaker adaptation from +500 μs and +1500 μs (weight of +1) adaptors. This contrast was not significant (p = 0.17). This indicates that the side from which a sound is perceived to originate is not a predictor of the extent to which it adapts cortical responses.

A similar pattern of results was observed for the ECoG data recorded from the cortex of the guinea-pig (Fig. 3d–e); responses to the −500-μs probe were smallest when the ITD of the adaptor was also −500 μs and largest when the adaptor was +500 μs. Further, both the −1500-μs and +1500-μs adaptors elicited a stronger adaptive effect than did the +500-μs adaptor. As for the human MEG data, this pattern is consistent with the presumed activation by the four adaptor ITDs in periodically tuned neurons (Fig. 3a; open circles). A repeated measures ANOVA with main effect of adaptor ITD was significant (F[3,9] = 13.3, p = 0.0012), as were both planned comparisons (periodic relation to stimulus centre-frequency: p = 0.0003; side of perceived laterality: p = 0.02). However, the significance for the first planned comparison—adaptors are stronger for periodically related ITDs - was two orders of magnitude stronger than for the second comparison (p = 0.0003 vs. p = 0.02), indicating that the periodic relationship between ITDs provided a better description of the data than did the side to which the sound was leading in time.

Despite their very different head-sizes, and therefore ethological range of ITDs, the data are highly consistent across the two species. Importantly, in terms of global cortical-activity patterns, the maximal release from adaptation, interpreted as the maximal separation of neural representations, occurred for an adaptor ITD maximally separated from the probe in terms of interaural *phase* difference (+500 μs – a separation of 180° with respect to the stimulus center-frequency) and with opposite perceived laterality, rather than for the adaptor most separated in terms of ITD (+1500 μs) and also of opposite perceived laterality.

### Periodic representation of ITD in human cortex generalizes across frequencies

One potential confound involving noise bands centered at 500 Hz is that the ITDs corresponding to ¼ and ¾ of the stimulus period at this frequency also lie within and beyond the ethological range of ITDs in humans. To this end, differences in adaptation generated by different ITDs might arise because, for example, the probe (−500 μs) and the least adapting ITD (+500 μs) lie within the human ethological range (±700 μs), whilst ±1500 μs, which generate intermediate adaptation, lie beyond the ethological range (see Fig. 3a). This issue is addressed in part by the similarity between our two data sets despite the different head sizes and ethological ranges of the two species. For the guinea pig, all of the stimulus ITDs lie beyond ethological range and yet, the pattern of adaptation in the guinea pig ECoG data was highly similar to that found in human MEG. This suggests a cortical representation of ITD dominated by the periodic relationship of ITDs, rather than the size of the head.

Nevertheless, in order to establish the existence of a periodic representation of ITD in cortical responses, it is critical to demonstrate its existence for frequencies other than 500 Hz, including for frequencies at which the potential confound between ITDs lying within or beyond the ethological range does not arise. We therefore assessed the same adaptation paradigm as at 500-Hz, for a band-pass noise centered at 1100 Hz, for two different ITD configurations (Fig. 4). The first configuration replicated the initial paradigm in which probe and adaptor ITDs are periodically related with respect to the stimulus center-frequency, but with all ITDs now lying within the human ethological range (Fig. 4a). For 1100 Hz, this corresponds to a probe ITD of −227 μs (-¼ cycle IPD) and adaptor ITDs of ±227 μs (±¼ cycle) and ±682 μs (±¾ cycle). The second configuration applied those ITDs corresponding to ¼ and ¾ cycle delays at 500 Hz (±500 μs and ±1500 μs, respectively) to the 1100-Hz centered stimulus, i.e. ITDs that are not periodically related at this center frequency but lie either within or beyond the human ethological range (Fig. 4c).

As for the 500-Hz centered stimulus, the 1100-Hz stimuli containing periodically related ITDs generated a pattern of activity in the MEG responses consistent with the presumed periodic activation by the adaptor ITDs (Fig. 4a, open circles); i.e. consistent with ITDs separated by a full period of the stimulus center-frequency having a shared neural representation. The N1 amplitude was modulated by the adaptor ITD (Fig. 4b; repeated-measures ANOVA with adaptor condition as repeating factor, F[3,27] = 3.25 p = 0.037), with responses to the −227-μs probe—equivalent to ¼ cycle leading at the left ear—most strongly adapted by sounds with the same ITD (as expected), and least adapted by an ITD of +227 μs, i.e. separated by half a cycle of the stimulus period and lateralized perceptually to the opposite side. Adaptation was intermediate for ITDs of −682 and + 682 μs. In other words, the smallest overlap of neural representations occurred when the ITD separation corresponded to the maximum IPD of half a cycle rather than when it was maximal in absolute terms. This generated a significant result in the first planned comparison testing for the period relation of ITD as a predicting factor (p = 0.0082). The second comparison testing for adaptation determined by perceived side of laterality was not significant (p = 0.22), consistent with the 500-Hz data.

In contrast, when ITDs corresponding to ¼ and ¾ cycle of the 500-Hz center frequency were applied to the 1100-Hz centered stimulus, the N1 amplitude again varied depending on the adaptor ITD (Fig. 4d; repeated-measures ANOVA with adaptor condition as repeating factor, F[3,27] = 5.32 p = 0.029), but the pattern of adaptation was markedly different from when ITDs equivalent to ¼ and ¾ cycles of the 1100-Hz center-frequency were applied. Predictably, responses to the −500-μs probe were most adapted when the ITD of the adaptor was −500 μs, but the least adaptive ITD was not +500 μs, as for the 500-Hz center-frequency, but, rather, +1500 μs. Intermediate adaptation followed from the −1500-μs and +500-μs stimuli. Unlike at 500 Hz, these ITD separations do not correspond to delays separated by a full period of the 1100-Hz stimulus center-frequency. Nevertheless, as for the periodic data at 1100 Hz, and at 500 Hz in both humans and guinea pigs (Fig. 3) the magnitude of the probe response is consistent with the presumed activation by the four adaptor ITDs (Fig. 4c; open circles on periodic function). Accordingly, the planned comparison corresponding to adaptation determined by the stimulus cycle at 500 Hz was not significant (p = 0.5), but the comparison for adaptation based on lateralization (p = 0.006) explains the data well. This outcome can still be understood intuitively in terms of stimulus periodicity at the center frequency. The −500-μs and +500-μs adaptors are closer to being separated from the probe ITD by a full stimulus cycle than is the +1500-μs adaptor that generates the least adaptation (see Fig. 4c). Together, these data suggest that the specific periodic relationship between ITDs within different sound-frequency channels is an important factor when interpreting cortical activity generated by sounds containing ITDs.

### A neural model explains adaptive effects of ITDs determined by stimulus center frequency

To test the intuitive interpretation that the cortical representation of ITD is periodic, we employed a cross-correlation model consisting of three stages: standard peripheral filtering by monaural elements prior to binaural integration, cross-correlation to model the activity induced by each adaptor and probe in a set of neurons selectively tuned to ITD and frequency, and an adaptation stage to model the influence of the adaptor on the probe response. To explore the ability of a restricted set of internal delays (preferred or best ITDs of neural elements) to account for the cortical data, we applied different weights to the cross-correlogram to simulate different distributions of best ITDs within each frequency band. Two distributions were considered, first, a homogeneous distribution of ITD detectors, but constrained to the π-limit (the upper bound of ½ a cycle re. stimulus centre frequency suggested by *in vivo* studies), and second, the Stern-Shear distribution of established psychoacoustic models in which the range of best ITDs extends well beyond the ethological range but with strong weighting to ITDs near zero. The distributions of best ITDs and an example of model activity for each of the 500-Hz stimuli before the adaptation stage is depicted in Fig. 2.

**Fig. 5:**
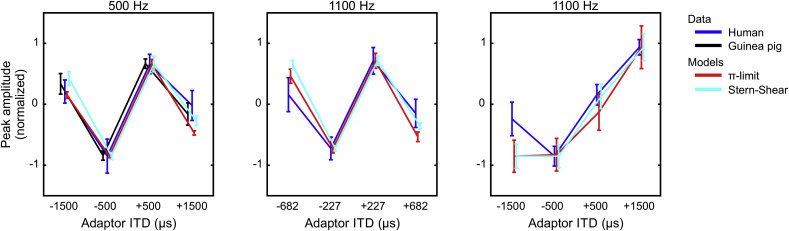
Predicted response amplitudes (average ± standard error of the mean over ten repetitions) from the computational model for the two distributions compared to the MEG data from humans and ECoG data from the guinea pig (plotted as in Fig. 3, Fig. 4). For illustration, peak amplitudes of the model predictions and ECoG data are scaled to have the same maxima and minima as the MEG data.

For the 500-Hz centered stimulus, the model predictions captured extremely well both the human MEG data and the guinea-pig ECoG data (Fig. 5). Overall, the predictions varied little between the two distributions of best ITDs (all model fits p < 0.01). This is perhaps not surprising, as a common feature of these models is their weighting of the distribution of internal delays, which favors ITDs relatively close to zero (central weighting) and within the π-limit. Model predictions were also derived for the 1100-Hz centered stimulus for probe and adaptor ITDs presented in periodic relationship to the center frequency (±227 μs and ±682 μs), as well as for probe and adaptor ITDs identical to those used in the 500-Hz frequency channel (±500 μs and ±1500 μs) and, therefore, *a*periodic in the 1100-Hz channel. In both cases, the predictions derived from the cross-correlation model resulted in a significant fit to the variation in the amplitude of the probe response as a function of the adaptor ITD (all model fits: p < 0.01).

## Discussion

We investigated the neural representation of ITD in the cortex of humans and guinea pigs, employing population-level recordings of neural activity in both species, and stimulus parameters identical to those used to motivate influential computational models of ITD processing (Trahiotis and Stern, 1989, Stern and Shear, 1996). Using an identical adaptor-probe paradigm, we found the neural representation of ITD at the cortical level to be determined by the center frequency of the sound and the periodic relations between ITDs with regard to the center frequency, rather than by the magnitude and sign of the ITD, or the range of ITDs permitted by the size of the head. In this sense, the data are consistent with the frequency-dependent and periodic tuning to ITD described in many animal studies of brainstem and midbrain auditory nuclei (reviewed in Grothe et al., 2010), and suggest that some aspects of human ITD processing at the subcortical level can be inferred from our understanding of data obtained from *in vivo* recordings at the cortical level. A relatively simple computational model implementing the π-limit, or a range of internal delays applied to the primary stage of binaural processing in human psychoacoustic models (and commonly thought to reflect neural activity in the lower brainstem; Stern et al., 1988), accounted well for the data.

The similarity between human MEG and guinea-pig ECoG data is intriguing because of the considerable difference in the ranges of ITDs experienced under natural listening conditions. This factor might have been expected to be important in neural coding of ITD, especially as ECoG recordings in the guinea pig were generated by ITDs that lie well beyond this species’ ethological range. Nevertheless, the strong correspondence between the data sets suggests a similar, potentially species-independent, representation of ITDs, evident even at the cortical level—one based on a common representation of interaural *phase* differences—instantiated similarly in each sound-frequency channel, consistent with *in vivo* recordings from a range of small mammals in the brainstem and midbrain (McAlpine et al., 2001, Brand et al., 2002, Franken et al., 2015).

### Is the neural representation of ITD based on time or phase?

Our observation that the neural representation of ITD in the auditory cortex is dependent on stimulus frequency, and seemingly determined by the stimulus IPD rather than ITD *per se* is perhaps surprising, considering how ITD is generated by a difference in arrival time of the sound at the ears and used in sound source localization. The azimuthal locations of real sources along the horizontal plane are therefore better related to ITD, rather than IPD (spherical head model measurements by Feddersen et al., 1957; but see also Kuhn, 1977, Benichoux et al., 2015, showing considerable frequency-dependence of ITD in more realistic measurements). Based on this, one could expect the brain to convert any IPD representation at the brainstem level into a frequency-independent representation of ITD in the cortex—one more closely related to how ITD relates to the azimuthal angle held by a sound source. Processing mechanisms are included in models that seek to account for headphone-based psychoacoustic performance, including operations that enhance neural detection of long ITDs (i.e. those beyond the π-limit) to account for correct lateralization judgements (Shackleton et al., 1992, Stern and Trahiotis, 1997). Nevertheless, our cortical data suggests that human auditory cortex (at least in terms of neural activity accessed by measures such as MEG) retains the periodic representation of ITDs evident in recordings from subcortical nuclei in small mammals. Since the N1 response primarily reflects activity from secondary rather than primary regions of the auditory cortex (Jääskeläinen et al., 2004), it appears unlikely that further conversions would take place at later stages. Also, it is unlikely that the adaptation effects recorded here would originate from subcortical nuclei rather than a cortical representation of ITD. The time-scale of our stimulus presentation was such that significant adaptation over such long inter-stimulus intervals occurs only cortically (Bartlett and Wang, 2005, Werner-Reiss et al., 2006). Therefore, whatever transformation in the neural code for binaural cues occurs between midbrain and cortex, the process is such that a periodic representation of ITD is still evident in cortically generated neural activity.

### Comparisons with previous studies—a cortical transformation in neural coding of ITD

Comparisons with previous studies of ITD sensitivity in human cortex are difficult, due to the sheer range of stimulus parameters employed. If stimulus features such as center-frequency, bandwidth and periodic relationship of ITDs are critical to the interpretation of cortical activity patterns, then interpreting data from studies employing stimulus parameters that depart from the standard psychoacoustic parameters becomes difficult, at least in terms of exploring the neural representation of ITDs instantiated in the human brain. Nevertheless, our MEG and ECoG data are consistent with the two previous functional imaging studies in which identical stimulus parameters were employed (Thompson et al., 2006, von Kriegstein et al., 2008), and which reported a transformation in the cortical representation of ITDs from midbrain to cortex in the blood oxygen level dependent (BOLD) response. ITDs within the π-limit (±500 μs in the 500-Hz frequency band) activated more the midbrain (inferior colliculus—IC) contralateral to the perceived location of the sound, whilst the longer ITDs beyond the π-limit (±1500 μs) activated more the IC ipsilateral to the perceived location (Thompson et al., 2006), consistent with the inherent periodicity of ITD processing, and with the operation of a π-limit. However, this pattern appears to undergo a transformation between midbrain and cortex, with long ITDs (>π) activating both cortices equally for the same listeners in the same recording sessions (von Kriegstein et al., 2008). Contrasted against each other, responses to long delays of equal ITD magnitude but opposite sign (±1500 μs) generated no significant voxels in either cortical hemisphere. Contrasted against zero ITD, however, both long ITDs showed significant, and bilaterally matched, activation. The current data suggest that some element of these long ITDs—of equal magnitude but opposite sign—might be important in generating a similar degree of adaptation (see Fig. 3, Fig. 4). One plausible explanation is that these long ITDs are identical in terms of their interaural correlation (IAC), the extent to which the sounds at both ears are similar. For headphone-listening tasks employing noise-bands, listeners are exquisitely sensitive to reductions in IAC (Pollack and Trittipoe, 1959, Gabriel and Colburn, 1981, Boehnke et al., 2002), including those generated by increasing magnitude of ITD. Reductions in IAC arise in natural listening through reflections from walls and other hard surfaces, or from multiple simultaneous sound sources, and contribute to the percept of the listening environment rather than source localization *per se*. Consistent with psychoacoustic performance (e.g. Gabriel and Colburn, 1981), the BOLD response in auditory cortex is sensitive to small changes in IAC, especially from a reference IAC of 1.0 (for a noise band with zero ITD; Budd et al., 2003). Given the importance of supra-ecological ITDs in generating the percept of the listening space (Traer and McDermott, 2016, Teng et al., 2017), it may be that cortical activity (or at least that accessible through whole-brain recordings) is dominated by the reduction in IAC generated by long ITDs, rather than by a lateralization percept that, whilst clearly perceived as lateralized to one side or the other, is only made possible under headphone-listening conditions.
